# Film and digital periapical radiographs for the measurement of apical root shortening

**DOI:** 10.4317/jced.50872

**Published:** 2012-12-01

**Authors:** Ahmed M F. El-Angbawi, Grant T. McIntyre, David R. Bearn, Donald J. Thomson

**Affiliations:** 1 BDS, MSc. PhD student, Orthodontic Department, Dundee Dental Hospital and School, University of Dundee, Dundee, DD1 4HR, UK; 2 BDS, FDS RCPS, MOrth RCS, PhD, FDS(Orth) RCPS, FDS RCS. Consultant / Honorary Senior Lecturer, Orthodontic Department, Dundee Dental Hospital and School, University of Dundee, Dundee, DD1 4HR, UK; 3 BDS, MSc, PhD, FDS(Orth) RCPS, MOrth RCS, FHEA. Professor / Honorary Consultant, Orthodontic Department, Dundee Dental Hospital and School, University of Dundee, Dundee, DD1 4HR, UK; 4 BDS, FDS RCS, DDR RCR Consultant / Honorary Senior Clinical Teacher, Department of Dental & Maxillofacial Radiology, Dundee Dental Hospital and School, University of Dundee, Dundee, DD1 4HR, UK

## Abstract

Objectives: The aim of this study was to compare the accuracy and agreement of scanned film and digital periapical radiographs for the measurement of apical root shortening. Study design: Twenty-four film and digital [phosphor plate sensor (PPS)] periapical radiographs were taken using the long-cone paralleling technique for six extracted teeth before and after 1mm of apical root trimming. All teeth were mounted using a typodont and the radiographs were recorded using a film holder and polysiloxane occlusal index for each tooth to ensure standardization during the different radiographic exposures. The film radiographs were scanned and the tooth length measurements for the scanned film and digital (PPS) images were calculated using Image-J-Link 1.4 software (http://rebweb.nih.gov/ij/index.html) for the two groups. The accuracy and agreement among the tooth length measurements from each group and the true tooth length measurements were calculated using intra-class correlation (ICC) tests and Bland and Altman plots. Results: A high level of agreement was found between the true tooth length measurements and the scanned film measurements (ICC=0.979, limit of agreement 0.579 to -0.565) and the digital (PPS) radiograph measurements (ICC= 0.979, limit of agreement 0.596 to -0.763). Moreover, a high level of agreement was found between the scanned film and digital (PPS) radiographs for the measurement of tooth length ICC=0.991, limit of agreement 0.411-0.231. Conclusion: Film and digital (PPS) periapical radiographs are accurate methods for measuring apical root shortening with a high level of agreement.

** Key words:**Root shortening, measurement, periapical radiographs, film, digital.

## Introduction

Orthodontically induced inflammatory root resorption (OIIRR) is a frequent but undesirable consequence of orthodontic treatment (1). Apical root shortening in the maxillary incisor region is usually the most evident manifestation of OIIRR; and the gold standard method for detecting and measuring root shortening during orthodontic treatment is periapical radiography (2). Conventional film periapical radiographs have been used for almost a century with developments in film speed and collimation improving image quality and minimising dose. With the introduction of digital imaging in 1986, digital periapical radiography became an alternative image modality (3).

Over the last 25 years, digital radiographic developments have led to improved image quality, reduced working time from image capture to display and reduced radiation dose to patients (3,4). In contrast to a radiographic film, digital imaging requires either a wired sensor placed in the patient’s mouth or a phosphor plate sensor (PPS) to temporarily store the radiographic energy of the latent X-rays. The latter is scanned before the radiographic image can be displayed on-screen. Despite the availability of digital radiography, conventional radiographic film is still commonly used as it is an inexpensive and reliable image receptor (5).

An alternative option to a fully digital system is to convert conventional film radiographs to digital images by scanning (6). This allows image quality to be enhanced (when necessary) and the images can be quantitatively analysed using on-screen software (7). However, it has been suggested that valuable diagnostic information can be lost during the digitisation procedure, with artefacts and noise being introduced (8). In addition, the radiation dose for the patient and the working time are not reduced as the radiographic technique is not altered.

Several experimental studies have compared the accuracy of diagnosing simulated external root resorption between conventional and digital radiographs (9-12). Westphalen et al (10) found that conventional film was inferior to digital peripical radiographs for the detection of simulated root resorption. Interestingly Levander et al (11) and Borg et al (12) reported that conventional film and digital periapical images had a similar level of sensitivity for the detection of resorption, but up to one quarter of lesions were not detected on either the conventional film or digital periapical radiographs. Kamburoglu et al (9) on the other hand determined that the presence of simulated apical root resorption was more difficult to identify using either conventional film or digital periapical radiographs in comparison to simulated resorption cavities elsewhere on the root surface. No study has yet compared the accuracy and validity of measuring simulated orthodontically induced apical root resorption using film and digital periapical radiographs.

The objective of this investigation was therefore to compare the accuracy and validity of scanned film and digital (PPS) periapical radiographs for the measurement of apical root shortening.

The null hypotheses tested were as follows:

1. Scanned film and digital (PPS) periapical radiographs are not accurate methods for measuring apical roo

shortening.

2. There is no difference between the measurements of tooth length between scanned film and digital (PPS)

periapical radiographs.

## Material and Methods

A sample size calculation determined that in order to be able to detect a clinically significant difference of 0.5 mm in tooth length between the two groups at a power of 80 percent where p<0.05, six sound extracted maxillary incisor teeth would be required. These were collected from the Oral Surgery Department at Alexandria University, Egypt and were judged to be caries free by observation with no obvious root defects. The teeth were sterilized and stored in 10 % formalin in a sealed container.

The true length of each of the six incisors was measured from the tip of the root to the midpoint (mesiodistally) of the incisal edge. The measurements were performed using digital calipers [Mitutoyo digital calliper (Mitutoyo.co.uk)] equipped with a Vernier scale accurate to 0.01 mm. The teeth were measured on two separate occasions and the mean length of each tooth was taken as the pre-trimming true length (PreT-TL).

Each tooth was placed into the central maxillary incisor region in a typodont (consisting of acrylic teeth in a wax base used for orthodontic fixed appliance training). Heavy body polysiloxane (Lab Putty, www.coltene.com) was used with a radiographic film holder for each mounted tooth to construct an index for the tooth for all four radiographic exposures of each tooth. Whilst the extracted incisor tooth was not fixed, it could be repositioned in a reproducible manner. A single small metal rod was placed in the wax of the typodont, mesial to each mounted tooth, for calculation of the magnification factor.

- Radiographs

Long-cone periapical radiographs were recorded (60 kv and 7 mA Dc, 0.20 sec) for each tooth in the typodont using the film holder polysiloxane index on two separate occasions by a single experienced dental radiographer, using a conventional film radiograph [F speed film (www.carestream.com)] and a digital (PPS) radiograph [Dürr Dental (www.duerr.co.uk)]. The distance of the source of the x-ray to the film was standardized at 40 cm.

The teeth were then removed from the typodont. All teeth had the root apex trimmed by 1mm using a new tungsten carbide bur in a slow speed dental hand piece. Each tooth was then measured using the same digital caliper twice and the mean length was taken as the post-trimming true length (PostT-TL).

The teeth were then remounted using the film holder index and two further long cone periapical radiographs (conventional and PPS) were taken for each tooth using the same technique as before.

- Measurements on the radiographs

The conventional radiographs were then digitised using a flatbed scanner [Epson perfection v750PRO (www.epson.com)] as 16 bit gray scale images at 300 dpi and no automatic adjustments. Both the scanned film and digital (PPS) periapical images were imported into Image J Link 1.4 software (http://rebweb.nih.gov/ij/index.html) for measurement. The total length of each tooth was measured from the apex of the root to the midpoint (mesio-distally) of the incisal edge. The true length of the metal rod and the length of the metal rod on the radiograph were used as a correction factor for the magnification to calculate the length of the tooth on the radiographs in millimeters. Each radiograph was measured by two observers (AE and GM), separately. Measurements were recorded on a second occasion two weeks later by one of the observers (A.E).

- Statistical analysis

. Intra-class correlation (ICC) was used to determine the inter-observer and intra-observer reliability.

. The level of accuracy of the two radiographic groups was evaluated in relation to the true length using ICC and Bland and Altman plots.

. The level of agreement between the two groups was determined using ICC and Bland and Altman plots.

. The ability to detect the change in tooth length was determined using paired t-rests for each group. The level of significance was set at P<0.05.

## Results

The mean true tooth length measurements of the teeth pre-trimming and post-trimming are listed in table (1).

- Intra-observer and inter-observer reliability

A high level of intra-observer and inter-observer agreement (ICC 0.978-0.997; reliability coefficient 0.992-0.999) was found for the measurements obtained from both scanned film and digital (PPS) radiographs.

- Accuracy and agreement

**Figure 1:**
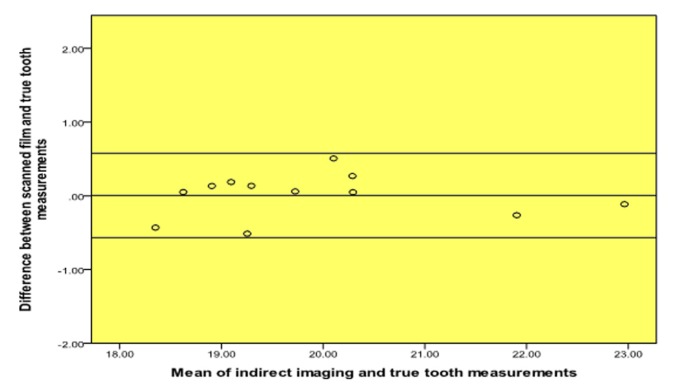
Bland and Altman plot of true tooth length vs. scanned film measurements.

**Figure 2:**
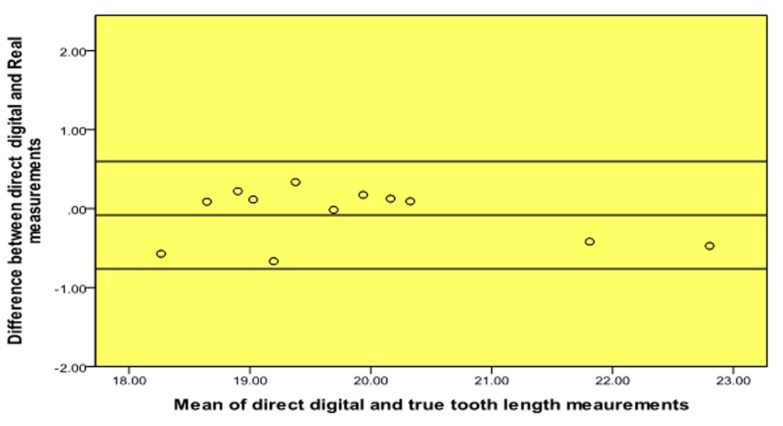
Bland and Altman plot of true tooth length vs. digital (PPS) measurements.

**Figure 3:**
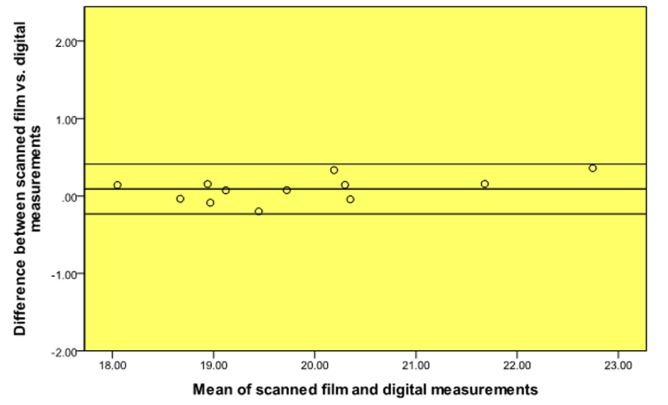
Bland and Altman plot of scanned film vs. digital (PPS) measurements.

A high level of agreement was found between the scanned film and digital (PPS) periapical radiographs and the true length of the teeth ( Table 1, Fig. 1, 2). Moreover, a high level of agreement was found between the measurements from the scanned films and digital radiographs (Table  Table 2, Fig. 3).

**Table 1:**
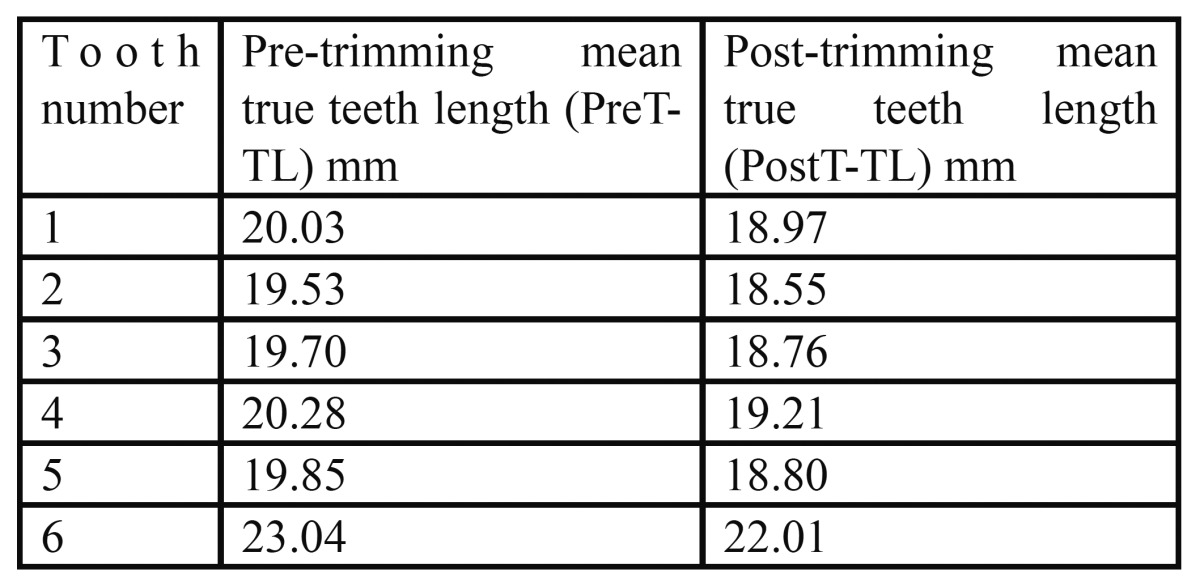
Mean true length measurements in millimeters of the experimental teeth before and after trimming using a digital caliper.

**Table 2:**
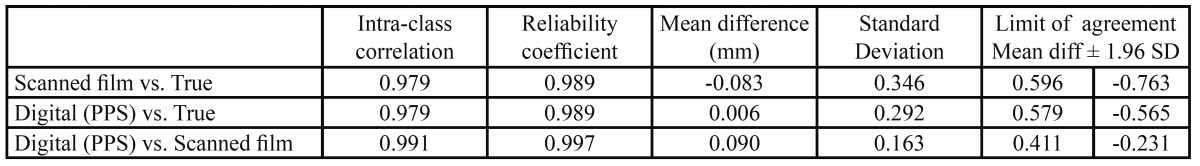
Measurements from the scanned films, digital (PPS) radiographs and the true length of the teeth.

- Radiographic measurements of apical root shortening

The pre- and post- shortening measurements of the six teeth for each observer for conventional film measure-ments and digital (PPS) measurements were analyzed with paired t-tests to determine if the radiographs were able to detect a 1 millimeter change in root length at a statistically significant level ( Table 3). In all cases the change in tooth length was statistically significant (P<0.01).

**Table 3:**
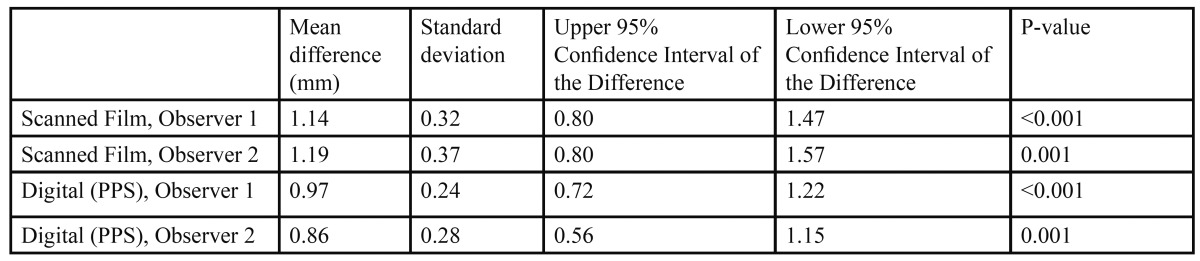
Mean measurements and results of paired t-tests for tooth shortening.

## Discussion

The objective of this study was to compare the accuracy of scanned film and digital (PPS) radiographs for the measurement of tooth length before and after apical root shortening. We found that scanned film and digital (PPS) radiographs are accurate methods for measuring tooth length and moreover, there were high levels of agreement between scanned film and digital (PPS) periapical radiographs. The difference in the means between the two groups (0.16 mm) was clinically insignificant (11), and suggests that scanned film and digital (PPS) periapical radiographs are both appropriate for the measurement of OIIRR.

Apical root shortening was done to simulate apical OIIRR in the current study by trimming the root apex by 1mm, which is the level at which it becomes clinically significant (11). We found that both conventional film and digital (PPS) radiographs were able to statistically significantly (p<0.01) detect the 1 millimeter change with the sample size of six teeth. This supports the use of both these imaging modalities to detect changes due to OIIRR. In the current study apical root shortening was done to simulate apical OIIRR rather than resorption concavities as used by Kamburoglu et al (9), Westphalen et al (10), Levander et al (11), and Borg et al (12). This was because we aimed to investigate the accuracy of the different radiographic methods for the linear measurement of tooth length rather than the sensitivity of detecting root resorption using subjective scoring systems. Three studies have compared linear measurements of root length using film and digital radiographs for endodontic purposes and found that they were comparable (13-15). Although these studies were not designed to assess simulated root resorption, it is noteworthy that our findings are in accordance with their findings.

We carried out a sample size calculation to determine the number of teeth that would be required. As only six teeth were required we were concerned that this could lead to random error obscuring the actual difference between the measurements made on the scanned film and digital (PPS) images. For this reason, we assessed the repeatability of the measurements for each group and compared the results to the true tooth length. Any systematic error could also bias the results and we aimed to minimize this by making the radiographs as standardized as possible. A silicon impression material index was used to customize the film holder for each study tooth to eliminate any difference in radiographic angulations between the study groups. Moreover, a single metal rod of known length was placed in the typodont close to the root of the experimental teeth to allow the magnification of each radiograph to be calculated.

In conclusion scanned film and digital (PPS) periapical radiographs are accurate methods for measuring tooth length with a high level of agreement.
